# CRISPR-*cas* Subtype I-Fb in *Acinetobacter baumannii*: Evolution and Utilization for Strain Subtyping

**DOI:** 10.1371/journal.pone.0118205

**Published:** 2015-02-23

**Authors:** Nabil Karah, Ørjan Samuelsen, Raffaele Zarrilli, Jason W. Sahl, Sun Nyunt Wai, Bernt Eric Uhlin

**Affiliations:** 1 The Laboratory for Molecular Infection Medicine Sweden (MIMS), Department of Molecular Biology, Umeå University, Umeå, Sweden; 2 Umeå Centre for Microbial Research, Umeå University, Umeå, Sweden; 3 Norwegian National Advisory Unit on Detection of Antimicrobial Resistance, Department of Microbiology and Infection Control, University Hospital of North Norway, Tromsø, Norway; 4 Department of Public Health, University of Naples “Federico II”, Naples, Italy; 5 CEINGE Biotecnologie Avanzate, Naples, Italy; 6 Department of Pathogen Genomics, Translational Genomics Research Institute, Flagstaff, Arizona, United States of America; St. Petersburg Pasteur Institute, RUSSIAN FEDERATION

## Abstract

Clustered regularly interspaced short palindromic repeats (CRISPR) are polymorphic elements found in the genome of some or all strains of particular bacterial species, providing them with a system of acquired immunity against invading bacteriophages and plasmids. Two CRISPR-Cas systems have been identified in *Acinetobacter baumannii*, an opportunistic pathogen with a remarkable capacity for clonal dissemination. In this study, we investigated the mode of evolution and diversity of spacers of the CRISPR-*cas* subtype I-Fb locus in a global collection of 76 isolates of *A. baumannii* obtained from 14 countries and 4 continents. The locus has basically evolved from a common ancestor following two main lineages and several pathways of vertical descent. However, this vertical passage has been interrupted by occasional events of horizontal transfer of the whole locus between distinct isolates. The isolates were assigned into 40 CRISPR-based sequence types (CST). CST1 and CST23-24 comprised 18 and 9 isolates, representing two main sub-clones of international clones CC1 and CC25, respectively. Epidemiological data showed that some of the CST1 isolates were acquired or imported from Iraq, where it has probably been endemic for more than one decade and occasionally been able to spread to USA, Canada, and Europe. CST23-24 has shown a remarkable ability to cause national outbreaks of infections in Sweden, Argentina, UAE, and USA. The three isolates of CST19 were independently imported from Thailand to Sweden and Norway, raising a concern about the prevalence of CST19 in Thailand. Our study highlights the dynamic nature of the CRISPR-*cas* subtype I-Fb locus in *A. baumannii*, and demonstrates the possibility of using a CRISPR-based approach for subtyping a significant part of the global population of *A. baumannii*.

## Introduction

Clustered regularly interspaced short palindromic repeats (CRISPR) are DNA elements present in the genome of 1176 out of 2612 fully sequenced bacterial strains (http://crispr.u-psud.fr/crispr/, last accessed December 2014). These elements together with neighboring genes, called *cas* for “CRISPR-associated”, provide bacteria with an adaptive immunity system against invading genetic elements, such as bacteriophages and plasmids [1]. The CRISPR-Cas immunity system functions in three steps: adaptation, expression, and interference [2]. Upon the entry of an invading element, the Cas machinery takes up a short sequence(s), a proto-spacer, from the invasive DNA and integrates it into the CRISPR array, where the adjacent direct repeat (DR) is duplicated and the integrated sequence becomes a new spacer. Next, the CRISPR array is transcribed and the produced RNA is cleaved and processed into small mature CRISPR RNAs (crRNA). Finally, the crRNAs guide the Cas nucleases for a sequence-specific cleavage of the invader. Flanking one side of the proto-spacers, a conserved short sequence, called PAM for Proto-spacer Adjacent Motif, has been identified [1]. PAMs, representing the recognition sites for the CRISPR-Cas machinery, play an important role in the adaptation and interference steps [3].

CRISPR—Cas systems are classified into three major types and various subtypes, based on the phylogenies of the conserved *cas* genes and the gene composition and architecture of the *cas* operons [4]. In addition, the CRISPR DRs are divided into at least 12 clusters, based on their sequence similarity and ability to form stable secondary structures [5]. Some of the DR clusters clearly correspond to particular CRISPR-Cas subtypes [4, 5]. Importantly, a linkage between the PAM sequences, typically 2–5 nucleotides long, and the main DR clusters has also been reported [3].

Generally, each CRISPR-*cas* locus includes a strain-specific array of spacers that has expanded and diversified over time [1, 6]. Due to their dynamic nature, comparative analysis of the arrays of spacers has successfully been used for subtyping isolates from several Gram-positive and-negative bacteria, including *Mycobacterium tuberculosis*, *Yersinia pestis* and the plant pathogen *Erwinia amylovora* (reviewed in [6]). Arrays of spacers were found to be highly polymorphic in *Salmonella* and a strong correlation was detected between polymorphisms in the arrays and the serotypes [7, 8]. In fact, analyzing only newly incorporated spacers gave results that were highly consistent with traditional serotyping of *Salmonella* isolates [8]. Furthermore, all the *S*. *enterica* Typhi and Paratyphi A isolates carried serotype-specific spacers that were exploited in the development of PCR assays able to identify these serotypes [7]. Other studies have focused on the evolutionary history of the CRISPR-Cas systems [9, 10]. For instance, frequent non-vertical transmission events have occurred throughout the evolution of the CRISPR-Cas system in *S*. *enterica* ssp. *enterica* [10].

*Acinetobacter baumannii* is an important opportunistic pathogen responsible for a wide range of hospital-acquired infections, including ventilator-associated pneumonia and catheter-related bloodstream infections [11]. Multilocus sequence typing (MLST), based on comparative sequence analyses of the loci of seven house-keeping genes, has demonstrated the frequent occurrence of *A*. *baumannii* isolates sharing similar allelic profiles although obtained independently from different countries [12, 13]. Using the MLST terminology, a “sequence type” (ST) refers to a particular allelic profile and “clonal complex” (CC) refers to a group of related STs sharing the same alleles at 5/7 or 6/7 of the loci (http://eburst.mlst.net). On the other hand, a “clone” is a general term that has been used to describe a group of phenotypically and genotypically related but epidemiologically unrelated isolates, which are believed to be a progeny of a common ancestor [14, 15]. Therefore, an ST or CC represents a “clone” only when it includes epidemiologically unrelated isolates, otherwise the terms are not interchangeable [14]. Two MLST schemes are currently available for *Acinetobacter* (http://pubmlst.org/abaumannii/). CC2, according to the Pasteur’s MLST scheme, is currently the largest and most widely distributed clone in the global population of *A*. *baumannii* [12, 13]. Nonetheless, several other clones have co-dominated or recently emerged as important international actors. For instance, CC1 ranks as the second largest clone of *A*. *baumannii*, with a broad international distribution in more than 30 countries from all continents [14]. Isolates from this clone have commonly showed a multidrug-resistance phenotype and frequently carried AbaR3-like resistance islands [16]. In parallel, a growing occurrence of CC25 has recently been reported from different countries in Europe, South and North America, Africa, and Asia [13, 14, 17, 18]. In addition to their extensive resistance to antibiotics, the CC25 isolates have shown the ability to resist desiccation, form biofilms on abiotic surfaces, and adhere to human alveolar epithelial cells [19].

Two CRISPR-Cas systems have recently been found in the genome of particular *A*. *baumannii* strains [20, 21]. The CRISPR-*cas* locus in strain AYE belongs to subtype I-Fb, herein denoted as CRISPR-*cas* subtype I-Fb throughout the manuscript [4, 22]. Genomic islands carrying this locus in strains 4190, AB0057 and AYE were found to be closely related, indicating potential inter-strain horizontal transfer [20]. Comparative analysis of partial sequences of the CRISPR-*cas* subtype I-Fb locus was useful in detecting the occurrence of an intra-clonal diversity among clinical isolates of international clone CC1 [21]. The aim of this study was to investigate the evolutionary history of CRISPR-*cas* subtype I-Fb in *A*. *baumannii* and to determine the genetic relatedness among a collection of CRISPR-positive clinical isolates of *A*. *baumannii*, based on comparative sequence analysis of the arrays of spacers located in their CRISPR-*cas* subtype I-Fb locus.

## Material and Methods

### A. baumannii *isolates*

The study included 74 isolates of *A*. *baumannii* carrying the CRISPR-Cas subtype I-Fb system (Table 1). The isolates were collected from the United States of America (USA; *n* = 29), Sweden (*n* = 12), Norway (*n* = 10), Iraq (*n* = 5), Netherlands (*n* = 3), Czech Republic (*n* = 3), Germany (*n* = 3), Canada (*n* = 2), Greece (*n* = 1), France (*n* = 1), Italy (*n* = 1), Argentina (*n* = 1), Colombia (*n* = 1), and United Arab Emirates (UAE; *n* = 1). The country of isolation was unknown for one isolate. Twenty-five isolates were part of three ongoing projects involving whole-genome sequencing of carbapenem-resistant *A*. *baumannii* isolates obtained in Norway between 2010 and 2013 (project I), Sweden between 2012 and 2013 (project II), or representatives of an international collection of *A*. *baumannii* isolates belonging to CC25 (project III). Forty-four isolates with sequenced genomes were selected from the records of the International Nucleotide Sequence Database Collaboration (http://www.insdc.org/). Occurrence of CRISPR-*cas* subtype I-Fb in these isolates was detected by Nucleotide BLAST algorithm (http://blast.ncbi.nlm.nih.gov/Blast.cgi), against both the “Nucleotide collection (nr/nt)” and “Whole-genome shotgun contigs (wgs)” databases. The remaining 5 isolates belonged to CC1 or CC25 and were part of previously published studies [23, 24]. The online multilocus sequence typing (MLST) service hosted by the Center for Genomic Epidemiology (http://www.genomicepidemiology.org/) in Denmark was used to determine the ST of all the isolates for which a full genome sequence was available [25]. The assignment was performed according to the Institute Pasteur’s MLST scheme [13] (http://pubmlst.org/abaumannii/). In order to group the isolates into CCs, a minimum spanning tree was generated from all the allelic profiles in the database using PhyloWeb and the MSTree application assimilated in the Institute Pasteur’s MLST web site (http://www.pasteur.fr/mlst).

**Table 1:** Epidemiological data on the 74 ***Acinetobacter baumannii*** isolates included in this study.

Isolate	Date and place of isolation^a^	Source and type of sample^a^	Import and other epidemiological data^a^	ST / CST^a^	GenBank accession no., [reference]
K48–42	Feb 2008, Unilabs Telelab, Skien, Norway	Tracheal aspirate	Import from India	ST1 / CST6	KM998765, [23]
K55–61	Mar 2009, Vestfold Hospital, Vestfold, Norway	Abdominal cavity	Import from India	ST94 / CST13	KM998766, [23]
K57–06	Mar 2009, Oslo University Hospital (Ullevål), Oslo, Norway	Trachael aspirate	Import from India, same PFGE pattern as K55–61	ST94 / CST13	[23]
AO-471	2005, Karolinska University Hospital, Stockholm, Sweden	Wound	Import from Thailand, tsunami-related	ST25 / CST30	KM998767, [24]
AO-21841	2006, Karolinska University Hospital, Stockholm, Sweden	Intra-abdominal	No history of import	ST25 / CST31	KM998768, [24]
K63–58	Mar 2010, Oslo University Hospital (Ullevål), Oslo, Norway	Cerebrospinal fluid	Import from Iraq	ST94 / CST12	KM998769
50509585	May 2011, Oslo University Hospital (Aker), Oslo, Norway	Trachael aspirate	Import from Greece	ST1 / CST8	KM998770
50525357	Jul 2011, Haukeland University Hospital, Bergen, Norway	Drain tube	Import from Romania	ST1 / CST9	KM998771
50535631	Sep 2012, Vestfold Hospital, Vestfold, Norway	Perineum	Import from Thailand	ST25 / CST20	KM998772
50678066	Dec 2012, Innlandet Hospital, Levanger, Norway	Tracheal	Import from Thailand	ST25 / CST19	KM998773
50691529	Feb 2013, Oslo University Hospital (Ullevål), Oslo, Norway	Urine	Import from Thailand	ST25 / CST19	Our unpublished data
50695882	Feb 2013, Sørlandet Hospital, Kristiansand, Norway	Urine	No history of import	ST1 / CST8	Our unpublished data
A068	Apr 2012, Blekinge Hospital, Blekinge, Sweden	-	-	ST25 / CST23	KM998774
A069	May, 2012, Halmstad Hospital, Halland, Sweden	Feces	Import from Thailand	ST25 / CST19	Our unpublished data
A076	Jan 2013, Skåne University Hospital, Skåne, Sweden	Rectum	-	ST1 / CST1	KM998775
A082	Mar 2013, Linköping University Hospital, Östergötland, Sweden	Wound	-	ST1 / CST4	KM998776
A092	Mar 2013, Linköping University Hospital, Östergötland, Sweden	Blood	-	ST25 / CST23	Our unpublished data
A093	Mar 2013, Linköping University Hospital, Östergötland, Sweden	-	-	ST25 / CST23	Our unpublished data
A094	Mar 2013, Linköping University Hospital, Östergötland, Sweden	Feces	-	ST25 / CST23	Our unpublished data
A096	Mar 2013, Linköping University Hospital, Östergötland, Sweden	Thorax	-	ST25 / CST23	Our unpublished data
A097	Mar 2013, Linköping University Hospital, Östergötland, Sweden	Nose	-	ST25 / CST23	Our unpublished data
A100	Oct 2013, Skåne University Hospital, Skåne, Sweden	-	-	ST1 / CST5	KM998777
RUH1486	1985, Rotterdam, Netherlands	Umbilicus	-	ST25 / CST29	KM998778, [12]
LUH7841	2002, Leiden, Netherlands	Intra-venous catheter tip	-	ST402 / CST15	KM998779
LUH6220	2000, Leiden, Netherlands	Sputum	-	ST25 / CST16	KM998780
4390	2003, Hippokration, Athens, Greece	Bronchial	Representing 3 isolates with the same PFGE pattern	ST25 / CST16	[40]
161/07	2007, Frankfurt University Hospital, Frankfurt, Germany	Respiratory tract	Import from Serbia	ST25 / CST28	KM998781, [41]
NM3	2008, Abu Dhabi, UAE	Sputum	Representing 4 isolates with the same PFGE pattern	ST25 / CST23	[39]
741019	2011, H7, Buenos Aires, Argentina	Pleural Fluid	Representing 7 isolates with the same PFGE pattern collected from 3 hospitals	ST25 / CST24	KM998782, [38]
4190	2009, Monaldi Hospital, Naples, Italy	Blood	Representing 3 isolates with the same PFGE pattern	ST25 / CST32	KM998783, [17, 20]
AYE	2001, Kremlin-Bicetre, France	Patient with pneumonia and urinary tract infection	Epidemic in 54 healthcare facilities in eight French administrative regions	ST1 / CST7	CU459141, [42]
AB0057	2004, WRAMC, Washington DC, USA	Blood	WRAMC was the major USA site receiving casualties from the conflict in Iraq/Kuwait and Afghanistan	ST1 / CST1	CP001182, [33]
Canada BC1	2007, a civilian hospital, Canada	-	Due to nosocomial spread of a war-related isolate introduced by a soldier evacuated via Landstuhl Regional Medical Center (Germany)	ST1 / CST1	AMSZ00000000
Canada BC-5	2007, a civilian hospital, Canada	-	Due to nosocomial spread of a war-related isolate introduced by a soldier evacuated via Landstuhl Regional Medical Center (Germany)	ST1 / CST1	AFDN00000000
IS-58	Feb 2008, Ibn Sina, Iraq	Respiratory tract	-	ST1 / CST1	AMGH00000000
IS-235	Aug 2008, Ibn Sina, Iraq	Blood	-	ST1 / CST1	AMEI00000000
IS-251	Sep 2008, Ibn Sina, Iraq	Respiratory tract	-	ST1 / CST1	AMEJ00000000
AB5075	2009, WRAMC, Maryland, USA	Bone infection	-	ST1 / CST1	AHAH00000000, [34]
AB_908–13	2007, Centers for Disease Control, USA	Urine	-	ST1 / CST1	AMHW00000000, [43]
AB_909–02–7	2007, Centers for Disease Control, USA	Sputum	-	ST1 / CST1	AMHZ00000000, [43]
TG20277	Jun 2006, Landstuhl Regional Medical Center, Landstuhl, Germany	Sputum	Isolated from a Canadian soldier injured in Afghanistan	ST1 / CST1	ASFH00000000
TG22112	2011, Arizona State Labs, USA	Trachael aspirate	-	ST1 / CST1	ASFK00000000
TG22148	2011, Arizona State Labs, USA	Trachael aspirate	-	ST1 / CST1	ASFN00000000
TG22190	2011, Arizona State Labs, USA	Trachael aspirate	-	ST1 / CST1	ASFP00000000
TG22194	2011, Arizona State Labs, USA	Trachael aspirate	-	ST1 / CST1	ASFR00000000
TG22196	2011, Arizona State Labs, USA	Blood	-	ST1 / CST1	ASFS00000000
TG22214	2011, Arizona State Labs, USA	Sputum	-	ST1 / CST1	ASFX00000000
1605	J. Craig Venter Institute, USA	-	-	ST1 / CST1	AUWL00000000
Naval-83	Oct 2006, National Naval Medical Center, Bethesda, Maryland, USA	Wound	-	ST20 / CST3	AMFK00000000
ABNIH19	2009, National Institutes of Health Clinical Center, Bethesda, Maryland, USA	Trachael aspirate	-	ST1 / CST2	APBH00000000, [44]
MRSN58	Jun 2010, WRAMC, USA	Wound	-	ST1 / CST2	JABU00000000
NIPH 290	Oct 1994, Příbram, Czech Republic	ICU	-	ST1 / CST2	APRD00000000, [37]
AB307–0294	1994, Buffalo, New York, USA	Blood	-	ST1 / CST10	CP001172, [33]
TG19582	American Type Culture Collection	-	-	ST1 / CST11	AMIV00000000, [43]
OIFC074	Jun 2003, Landstahl Regional Medical Center, Landstahl, Germany	-	-	ST19 / CST13	AMDE00000000
MRSN 3405	Mar 2011, WRAMC, USA	Wound	-	ST94 / CST13	JNOU00000000
NIPH 201	Jul 1992, Liberec, Czech Republic	ICU	-	ST38 / CST35	APQV00000000, [37]
ab233846	USA	Sputum	-	ST126 / CST38	JMOG00000000
ab299505	USA	Perirectal	-	ST508 / CST36	JEWY00000000
NIPH 615	Jan 1994, Praha, Czech Republic	Burns unit	-	ST12 / CST39	APOV00000000, [37]
TG27391	Arizona State Labs, USA	-	-	ST427 / CST40	ASGK00000000
ab532279	USA	Perirectal	-	ST519 / CST37	JEYH00000000
ab1106579	USA	Perirectal	-	ST505 / CST33	JEXN00000000
Naval-82	Oct 2006, National Naval Medical Center, Bethesda, Maryland, USA	Blood	-	ST428 / CST34	AMSW00000000
OIFC143	Jul 2003, WRAMC, USA	-	-	ST25 / CST25	AFDL00000000
abC179	Mar 2005, Iraq	Respiratory tract	From military personnel, combat-related	ST25 / CST26	AVOD00000000, [45]
abCI86	Feb 2005, Iraq	Superficial wound	From military personnel, combat-related	ST25 / CST26	AVOB00000000, [45]
ab984213	USA	Perirectal	-	ST25 / CST17	JEVX00000000
Naval-18	Jun 2006, National Naval Medical Center, Bethesda, Maryland, USA	-	-	ST25 / CST27	AFDA00000000
AB-1650–8	2006, Arizona State Labs, USA	Bone (from hip)	-	ST113 / CST14	AMHG00000000, [43]
ab1429530	USA	Perirectal	-	ST25 / CST21	JEWM00000000
107m	Colombia	-	-	ST25 / CST22	CBSG00000000
AB_2008–15–69	2008, Centers for Disease Control, USA	-	-	ST25 / CST18	AMHN00000000, [43]
AB5256	2009, WRAMC, Maryland, USA	Blood	Representing a clonal group	ST25 / CST23	AHAI00000000, [34]

^a^ ST, Sequence type; CST, CRISPR-based sequence type; PFGE, Pulsed-field gel electrophoresis; UAE, United Arab Emirates; WRAMC, Walter Reed Army Medical Center; USA, United States of America; ICU, Intensive care unit.

### Phylogenetic analysis

Phylogenetic trees were generated based on nucleotide sequences alignments of (i) a conserved segment of 101 bp located downstream of the array of spacers, (ii) 920 bp of the *cas1* gene, (iii) 1251 bp of the *csy1* (Cas system-associated) gene, (iv) 615 bp of the *csy4* gene, and (v) 2976 bp of the concatenated MLST sequences. Only isolates with sequenced genomes were included in the phylogenetic analyses. The online available package of programs (MUSCLE, Gblocks, PhyML, and TreeDyn) was used for nucleotide alignment, tree construction, and tree rendering [26]. One hundred bootstraps were used for bootstrap analysis.

### CRISPR-based subtyping

DNA sequences of the CRISPR arrays of spacers were either retrieved from the GenBank nucleotide database or amplified and sequenced using the BigDye 3.1 technology (Applied Biosystems). Amplification of the full length of the arrays was performed using a pair of external primers, Ab-CRIS-F and Ab-CRIS-R, targeting conserved flanking regions (S1 Table). PCR products were purified using ExoProStar (GE Healthcare Bio-Sciences). The purified PCR amplicons were subsequently sequenced using the two external and several internal primers designed in tandem as required. Arrays of spacers were identified using CRISPRFinder [27]. A dictionary of annotated spacers was created using CRISPRtionary (http://crispr.u-psud.fr/CRISPRcompar/Dict/Dict.php) and revised manually. CRISPRtionary was also used to create a binary file of presence (1) or absence (0) of spacers. Each spacer with a newly defined sequence was assigned a new consecutive number, and each array with a newly defined assortment of spacers represented a new CRISPR-based sequence type (CST), as previously described [28].

### GenBank Accession Numbers

Nucleotide sequences of the CRISPR arrays in isolates K48–42, K55–61, AO-471, AO-21841, K63–58, 50509585, 50525357, 50535631, 50678066, A068, A076, A082, A100, RUH1486, LUH7841, LUH6220, 161/07, 741019, and 4190 were deposited in the GenBank nucleotide database under accession numbers KM998765 to KM998783, respectively.

## Results and Discussion

### Description and distribution of the CRISPR-*cas* subtype I-Fb locus

**Fig 1:**
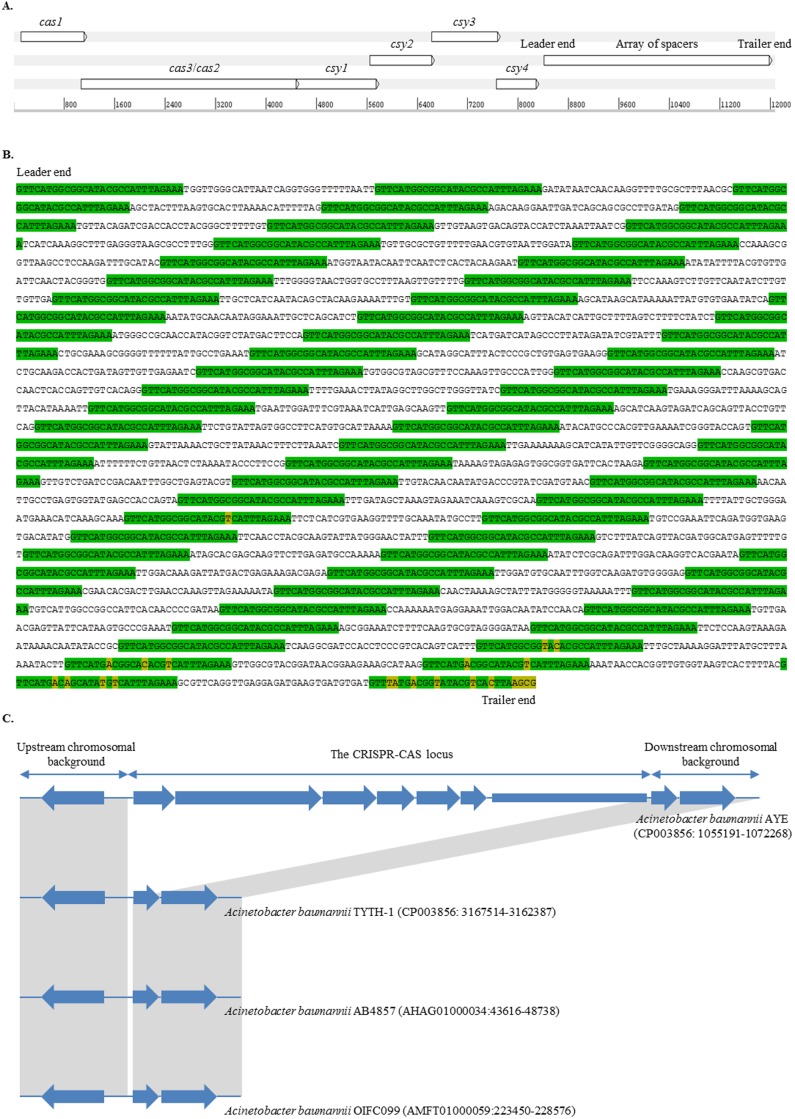
Genetic structure of the CRISPR-*cas* subtype I-Fb locus in *Acinetobacter baumannii*. *A)* The CRISPR-*cas* subtype I-Fb locus in *A*. *baumannii* strain AYE (GenBank accession number: CU459141, located at position 1,057,691 to 1,069,768). The locus consisted of two CRISPR–associated genes (*cas1* and *cas3*/*cas2*), four Cas system-associated genes (*csy1*, *csy2*, *csy3*, and *csy4*), and an array of spacers. The map was created using Artemis (http://www.sanger.ac.uk/resources/software/artemis/). *B)* Nucleotide sequence of the array of spacers in *A*. *baumannii* strain AYE. The array included 59 spacers surrounded by 60 direct repeats (marked in green). Some repeats, mainly at the trailer end of the array, included degenerated nucleotides (marked in yellow). *C)* Comparative analysis of the genetic surroundings. The comparison was performed between the locus-positive strain AYE which belonged to sequence type (ST1) and the locus-negative strains TYTH-1, AB4857, and OIFC099 which belonged to ST2, ST3, and ST32, respectively. Homologous sequences shared by all the isolates were indicated by gray zones.

The CRISPR-*cas* subtype I-Fb locus, located at position 1,057,691 to 1,069,768 of the genome of *A*. *baumannii* strain AYE (GenBank accession number: CU459141), consisted of six genes consecutively encoding the Cas1 endonuclease, Cas3/cas2 helicase/RNAse, and four Csy proteins (Fig. 1A and B). *csy4* (*cas6f*) was followed by a short non-coding TA-rich leader sequence. Then, the locus enclosed an array of spacers, where each spacer was flanked by two DRs. The spacers had variable sequences and a common length of 32 bp, whereas the DRs had the 5′-GTTCATGGCGGCATACGCCATTTAGAAA-3′ consensus sequence and were 28 bp long. Similarly to the CRISPR-Cas subtype I-F systems in other genera, the consensus sequence belonged to the DR cluster 4, showing a nucleotide similarity of 65–75% with those of *Escherichia coli*, *Pseudomonas aeruginosa*, *Yersinia pestis*, *Shewanella* spp., and *Pectobacterium atrosepticum* [3, 5, 29, 30]. Overall, 876 distinct *A*. *baumannii*-spacers (Ab-1 to-876) were identified (S2 Table). The size of the arrays was remarkably different among the isolates, ranging from 148 bp (2 spacers) up to 7354 bp (122 spacers).

A complete CRISPR-*cas* subtype I-Fb locus was present in all isolates belonging to CC1 and CC25. The locus was also present in isolates from ST113, ST12, ST38, ST126, ST427, ST505, ST508 and ST519. An internal deletion was detected in the locus of only one isolate, Naval-82 that belonged to ST428, resulting in the absence of *cas3*/*cas2*, *csy1*, *csy2* and part of *csy3* (GenBank accession number: AMSW01000159). On the other hand, the locus was replaced by a short sequence of 128 bp in the genome of *A*. *baumannii* strain TYTH-1 (GenBank accession number: CP003856), which was assigned to ST2 (Fig. 1C). The locus was also not present in the genome of other isolates from CC2 or isolates AB4857 and OIFC099 from the epidemic clones CC3 and CC32, respectively (Fig. 1C and data not shown). Notably, the locus was present in isolates from other *Acinetobacter* species such as *Acinetobacter haemolyticus* TG19602 and *Acinetobacter gyllenbergii* NIPH 230 (GenBank accession numbers: AMJB01000210 and AYEQ01000180, respectively). In addition, a common occurrence of CRISPR-*cas* subtype I-Fb in *Acinetobacter parvus* has recently been reported [22].

A Blastn search failed to detect proto-spacers with homologous sequences to 42/106 of the spacers present in *A*. *baumannii* CC1 isolates (S2 Table). This could be due to the limited number of *A*. *baumannii* phages that have been sequenced and deposited in the GenBank databases [22]. Nonetheless, phage- and plasmid-related DNA elements were the source of 50/106 and 12/106 of the spacers, respectively. The remaining 2/106 spacers originated from *A*. *baumannii* DNA that was most likely not related to phages or plasmids. These results were comparable to those of previous studies on the CRISPR arrays in *Streptococcus thermophilus* and *Y*. *pestis* [9, 31]. For example, only 500 out of 952 unique spacers in *S*. *thermophilus* showed similarity to viral (*n* = 384), plasmid (*n* = 80), or chromosomal (*n* = 33) sequences [9]. A prophage of 42778 bp, located on the genome of *A*. *baumannii* NIPH 527 (APQW01000004: 179581–222358), represented the main foreign DNA encountered by our isolates, being the source of 16/106 of the spacers (S2 Table and S1 Fig.). The proto-spacers in this prophage were carried on a variety of genes, such as the replicative DNA helicase, glycosyl hydrolase, tail tape measure, integrase, terminase, and GDSL-family lipase genes, or located in inter-gene regions. The proto-spacers were found either on the sense or antisense strand (S1 Fig.), as previously described [3].

Alignment of sequences surrounding the proto-spacers identified the dinucleotide “CC”, or “GG” on the complementary strand, to be the PAM for the CRISPR-Cas subtype I-Fb machinery of *A*. *baumanni* (S2 Table and S2 Fig.). The PAM motif was located on the 5′ end of the proto-spacers, as previously reported for other CRISPR-Cas type I systems [2]. Our results were also consistent with earlier studies showing that GG has been the signature PAM for the DR cluster 4 and CRISPR-Cas subtype I-F systems [3]. A set of 4 spacers (Ab-62 to Ab-65) was most likely acquired by one of our isolates, assigned to CST8, after a single contact with a plasmid from *A*. *baumannii* IS-58 assigned to CST1 (S2 Table). Similarly, Ab-77 to Ab-80, Ab-92 to Ab-94 (S1 Fig.), and Ab-97 to Ab-99 could also be acquired after single interactions with particular plasmid or phage DNA molecules.

### Evolution of the CRISPR-*cas* subtype I-Fb locus

Arrays of spacers have mainly evolved by adding new spacers in response to contacts with foreign genetic elements [9]. Since the addition takes place in a one-way direction, spacers present at one end (the trailer end) of the arrays had been integrated earlier than those present in the other end (the leader end), and the order of spacers generally provides a chronological narration of former exposures to invading phages and plasmids [31]. Studies have reported that trailer end spacers are generally conserved among different isolates and can be used to anchor clusters and detect common ancestors of the arrays and probably of the isolates themselves [9]. In contrast, the leader end spacers are usually polymorphic, reflecting the existence of distinctive phage/plasmid pools at a particular era in different geographic locations [8]. Ab-1 and Ab-107 were the first spacers acquired by the vast majority of our isolates (S2 Table). Spacer Ab-1 was present in the locus of all the isolates from CC1, ST38, ST428, ST505, and ST519, and also isolate 4190 from CC25. In contrast, spacer Ab-107 was shared by all the CC25 isolates, except isolate 4190, and the ST113, ST126, ST12, and ST427 isolates. The conservation of Ab-1 and Ab-107 at the trailer end of the arrays putatively assembled our arrays into two major groups with a different first step of descending from a common ancestor empty of spacers. In agreement with previous studies, the DRs at the trailer end of our isolates were degenerated (Fig. 1B). However, the number and sequence of the degenerated nucleotides were following two patterns corresponding to the Ab-1 and Ab-107 grouping of the isolates, which confirmed the occurrence of two main lineages in the early evolutionary history of the locus (S3 Fig.). Furthermore, comparative sequence analysis of a conserved segment of 101-bp located adjacent to the trailer end of the arrays showed a precisely matching assembly of the isolates (S4 Fig.).

**Fig 2:**
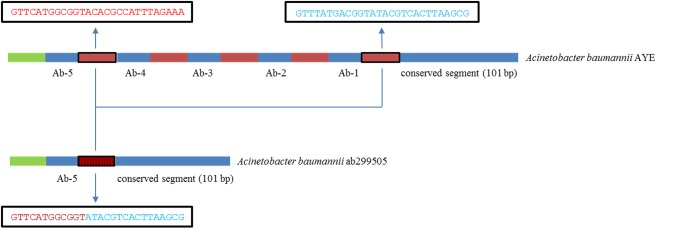
Schematic comparison of the trailer end of the arrays of spacers in *Acinetobacter baumannii* AYE and *A*. *baumannii* ab299505. The region carrying spacers Ab-1 to Ab-4 in *A*. *baumannii* AYE was missing in *A*. *baumannii* ab299505, most likely due to an internal deletion caused by a recombination event between the two direct repeats (highlighted by a black frame) surrounding the deleted region. This created a unique direct repeat (highlighted by a black frame and vertical lines) characterized by a novel mosaic sequence derived from the recombined direct repeats. Sequence of the direct repeats involved in the recombination was presented in adjacent black boxes.

The CRISPR arrays have frequently been reshaped by internal deletions and duplications, of individual spacers or sets of consecutive spacers, leading to further diversification of the arrays [32]. Interestingly, Ab-5 was located first at the trailer end of the array of spacers in isolate ab299505 (ST508). This was probably due to an internal deletion of 240 bp, erasing Ab-1 to Ab-4, caused by a recombination event between the two DRs surrounding the deleted region (Fig. 2). The last DR in this array had a unique sequence that was most likely derived from the two recombined DRs, consistent with the occurrence of such a recombination event. Accordingly, the locus in ab299505 also belonged to the Ab-1 lineage (S4 Fig.).

**Fig 3:**
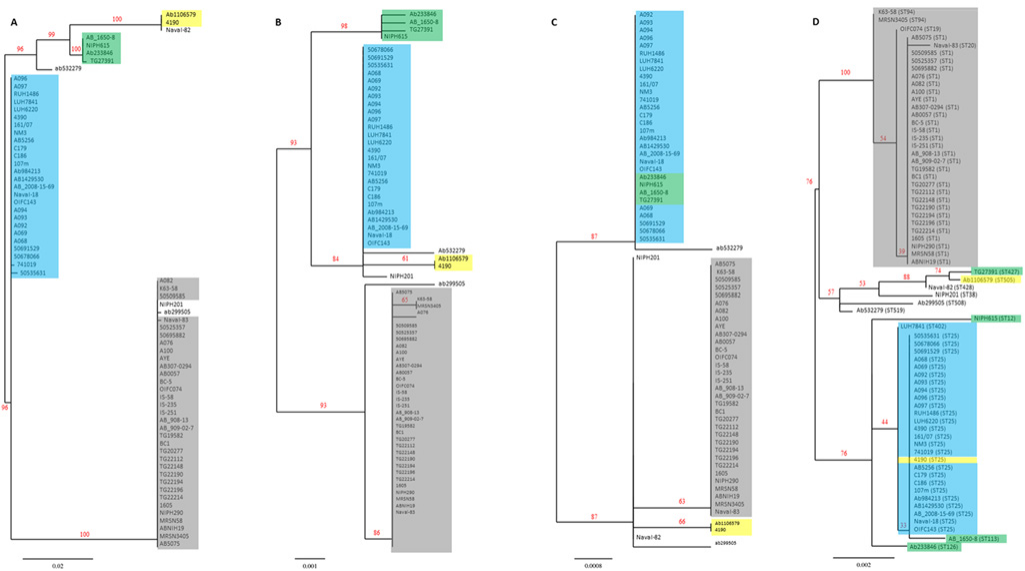
Phylogenetic trees of *cas1*, *csy1*, *csy4* and the concatenated MLST sequences. The phylogenetic trees were based on aligned nucleotide sequences of (*A*) 920 bp of *cas1* from 69 isolates of *Acinetobacter baumannii*, (*B*) 1251 bp of *csy-1* from 68 isolates of *A*. *baumannii* since *csy1* was lacking in isolate Naval-82, (*C*) 615 bp of *csy4* from 69 isolates of *A*. *baumannii*, and (*D*) 2976 bp of concatenated MLST sequences of 69 isolates of *A*. *baumannii*. MUSCLE, Gblocks, PhyML, and TreeDyn were used for nucleotide alignment and tree construction. One hundred bootstraps were used for bootstrap analysis. Branch support values were displayed in %. Isolates of the CRISPR-*cas* subtype I-Fb pathways of evolution 1, 2, 3, and 4 were highlighted in gray, blue, green, and yellow, respectively. The corresponding sequence type (ST) was presented next to the name of each isolate in (*D*).

Phylogenetic trees of *cas1*, *csy1*, and *csy4* identified seven distinct pathways of evolution for the CRISPR-*cas* subtype I-Fb locus in *A*. *baumannii* (Fig. 3A, B, and C). Pathways 1, 4, 5, 6, and 7 branched from the Ab-1 lineage whereas pathways 2 and 3 descended from the Ab-92 lineage. Pathway 1 ended with a cluster including all the alleles of the locus present in isolates from CC1. Similarly, pathway 2 created a cluster of all the alleles of isolates from CC25, except for strain 4190. Pathway 3 was shared by the alleles of isolates AB_1650–8 (ST113), NIPH615 (ST12), ab233846 (ST126), and TG23791 (ST427), whereas the two alleles of isolates 4190 (CC25) and ab1106579 (ST505) evolved following pathway 4. The alleles of isolates NIPH201 (ST38), ab299505 (ST508), and ab532279 (ST519) showed individual pathways of evolution. However, the latter three alleles had mosaic sequences proposing the occurrence of penetrations mediated by recombination events with the locus of other strains. Due to the internal deletion, the pathway of evolution could not be determined for the locus in Naval-82 (ST428). Comparing the topology of the isolates in these phylogenetic trees with the one based on the concatenated MLST sequences showed a congruent positioning of the isolates of pathways 1 and 2, suggesting a vertical spread of the locus in these two clusters (Fig. 3D). On the other hand, the incongruent standing of the isolates of pathways 3 and 4 proposed the occurrence of recent horizontal acquisitions of the whole locus between isolates of each cluster [10].

### CRISPR-based subtyping

**Fig 4:**
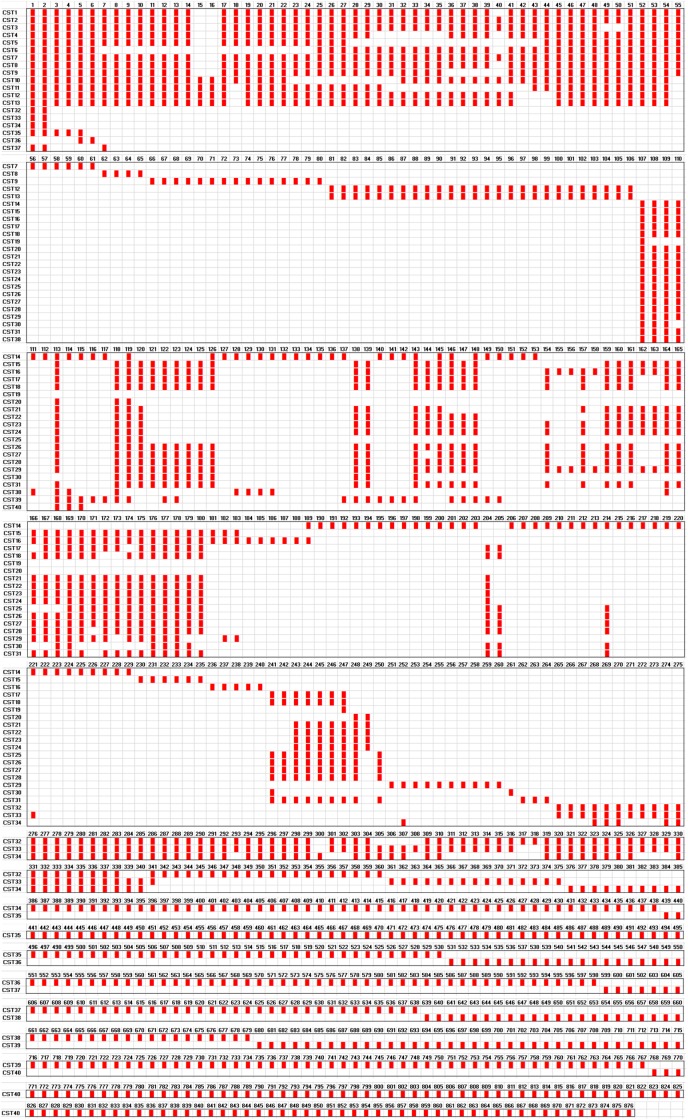
Graphic representation of the arrays of spacers in the CRISPR-*cas* subtype I-Fb locus of *Acinetobacter baumannii*. The figure demonstrated the assortment of 74 *A*. *baumannii* isolates into 40 CRISPR sequence types (CST) based on the spacer content of their CRISPR arrays. Spacers were represented by red rectangles. Each unique spacer was assigned a number (1–876). Spacers were sequentially aligned in order to facilitate comparison among the CSTs.

Different assortments of the spacers divided the isolates into 40 CSTs (Fig. 4 and S3 Table). Isolates from CC1 (*n* = 36) belonged to 13 CSTs, with some CSTs being different from each other only by a duplication or deletion of 1 spacer. CST1 included 18 isolates recovered between 2004 and 2013 from USA (*n* = 11), Iraq (*n* = 3), Canada (*n* = 2), Germany (*n* = 1), and Sweden (*n* = 1). Tracking the epidemiological data showed that eight of the isolates were obtained during the military operations in Iraq and Afghanistan [33, 34]. CST1 could be an Iraq-endemic sub-clone of CC1 that was able to spread to USA, Canada, and Europe. A previous study comparing the DNA profiles of *A*. *baumannii* isolates from USA and the United Kingdom that were associated with casualties returning from the Iraq conflict has also demonstrated the import of at least one strain responsible for outbreaks of infections in the two countries [35]. Adaptation of CST1 to the pool of phages and plasmids present in a particular geographical site resulted in the acquisition of specific spacers which might be used as a genomic signature of this sub-clone and a biological marker of this particular geographic ecosystem [10, 36]. On the other hand, CST2 included 3 isolates obtained from Czech Republic in 1994 and USA in 2009 and 2010. However, the Czech isolate was not reported to be epidemiologically linked with the two American isolates [37].

CST8 included two isolates obtained in 2011 and 2013 by two different diagnostic laboratories in Norway. Since no other epidemiological linkage was detected, inter-hospital transfer of patients or medical staff could be responsible for the spread of CST8. However, the long gap in the time of acquisition obviously excluded the occurrence of an outbreak. CST12 was different from CST13 only by having a duplication of spacer Ab-49. Five isolates belonged to CST12–13. Three of these isolates, obtained from Germany, Norway, and USA between 2003 and 2011, shared a history of import from Iraq (Table 1). The other two isolates were obtained from different laboratories in Norway in 2009 but were both imported from India [23]. Interestingly, none of these isolates belong to ST1, the founder of CC1. In contrast, the isolates mainly belong to ST94, indicating a considerable sub-clonal demarcation of ST94, able to overcome the origination of the isolates from two geographically disconnected countries. Three isolates, obtained from patients in one military hospital in France in 2009, were found to carry arrays of spacers with a high similarity to each other [21]. The three isolates were linked to CST12–13 according to our subtyping scheme. However, the comparison was incomplete since only the leader end of the CRISPR arrays was amplified and partially sequenced in the French isolates. Of note, the three French isolates were recovered from skin samples while our isolates came from various sources [21].

The CC25 isolates (*n* = 30) were divided into 19 CSTs (Table 1 and Fig. 4). Three isolates, obtained from Sweden in 2012 and Norway in 2012 and 2013, belonged to CST19. Interestingly, these isolates were recovered from patients previously hospitalized in Thailand. The molecular similarity between the isolates suggested the existence of a Thai strain and supported the history of import of these isolates, pointing once more to the necessity of having a screening program for patients after hospitalization abroad [23]. CST23 was identical to CST24 apart from having a duplication of three spacers. CST23–24 included 6 isolates obtained from Sweden and 3 isolates obtained from Argentina, UAE, and USA. The first Swedish isolate was collected in Blekinge in April 2012 while the other 5 isolates came from 5 different patients hospitalized at the same medical center in Östergötland in August 2013. The Swedish isolates, particularly the latter five, could represent a single strain responsible for a small-sized outbreak taking place in Östergötland. The Argentinean strain represents 7 isolates sharing the same pulsed-field gel electrophoresis (PFGE) pattern [38]. These isolates were recovered between 2009 and 2012, during an endemic setting in three hospitals located in two different cities in Argentina. Similarly, the UAE and USA strains are representatives of two groups of isolates sharing same PFGE patterns [34, 39]. The ability of CST23–24 to be endemic and to cause outbreaks of infections highlights the need to precisely distinguish such highly-successful sub-clones, which requires the application of more strict infection control procedures. Isolates from singleton STs (*n* = 8) belonged to 8 distinct CSTs. These isolates carried >80% of the unique isolate-specific spacers. The broad polymorphism detected among the spacers reflects the complicity and extensive diversity of phage and plasmid populations, facilitating the occurrence of several events of independent interactions and leading to separated pathways of evolution of the CRISPR arrays.

## Conclusion

Vertical transmission of the CRISPR-*cas* subtype I-Fb locus in our global collection of *A*. *baumannii* clinical isolates took place following two main lineages and several pathways of descent from a common ancestor. Occasional events of horizontal transfer have increased the diversification and facilitated further dissemination of the locus. Using the CRISPR-based subtyping approach, we were able to detect a sub-clone of *A*. *baumannii* CC1, probably originating in Iraq and spreading internationally to the USA and Europe. The study also detected a sub-clone of *A*. *baumannii* CC25 with a remarkable ability to cause outbreaks of infections. The unambiguous data generated by this approach can readily be exchanged *in silico*, used by other groups, and expanded by forthcoming projects. Overall, CRISPR-based subtyping supplements MLST and can be used to track the source and dissemination routes of particular strains.

## Supporting Information

S1 Fig Genetic structure of a prophage carrying 16 proto-spacers and representing an invader most frequently interacting with the CRISPR-Cas subtype I-Fb machinery of *Acinetobacter baumannii*. The prophage, 42778-bp long, was located on the genome of *A*. *baumannii* NIPH 527 (APQW01000004: 179581–222358). The prophage was shown as a white box on the graph and described as a “mobile_element” in the feature list. Genes and open reading frames were shown as blue arrows, with the arrowheads indicating the direction of transcription. Proto-spacers were presented as labeled green arrows, with the arrowheads indicating the direction of their integration as spacers in the CRISPR arrays. The prophage was surrounded by two identical 20-bp repeat regions, for which the sequences were indicated on the graph. The map was created using Artemis (http://www.sanger.ac.uk/resources/software/artemis/).
(TIF)

S2 Fig Sequence logo showing the proto-spacer adjacent motif (PAM) of the CRISPR-Cas subtype I-Fb machinery of *Acinetobacter baumannii*. The sequence logo was created based on an alignment of 10-bp sequences adjacent to 63 proto-spacers targeted by the CRISPR-Cas subtype I-Fb system of *A*. *baumannii*. The alignment and the logo were created using WebLogo (http://weblogo.berkeley.edu/logo.cgi). The logo consisted of stacks of letters, with a maximum height of 2 bits. The height of letters within each stack reflects the relative frequency of the corresponding nucleotide at that position. The alignment defined CC as the proto-spacer adjacent motif (PAM) for the CRISPR-Cas subtype I-Fb machinery of *A*. *baumannii*.
(TIF)

S3 Fig Comparative sequence analysis of the direct repeats present at the trailer end of the arrays of spacers. Pattern 1 was detected in all isolates from CC1, ST38, ST428, ST505, and ST519, and isolate 4190 from CC25. Pattern 2 was detected in all isolates from CC25, except for strain 4190, and in the isolates from ST113, ST126, ST12, and ST427. Preserved and degenerated nucleotides of the direct repeats were marked in green and yellow, respectively.
(TIF)

S4 Fig Phylogenetic tree of a conserved segment adjacent to the trailer end of the array of spacers of the CRISPR-*cas* subtype I-Fb locus. The tree was based on aligned nucleotide sequences of 101 bp from 69 isolates of *Acinetobacter baumannii*. MUSCLE, Gblocks, PhyML, and TreeDyn were used for nucleotide alignment and tree construction. One hundred bootstraps were used for bootstrap analysis. Branch support values were displayed in %. Isolates of linages Ab-1 and Ab-107 were indicated by braces.
(TIF)

S1 Table Primers for amplification and sequencing the arrays of spacers. (XLS)

S2 Table Distribution of spacers Ab-1 to Ab-876 among 74 isolates of *Acinetobacter baumannii* carrying the CRISPR-Cas subtype I-Fb system, and description of DNA elements targeted by the first 106 spacers. (XLS)

S3 Table Assortment of the Ab spacers into forty CRISPR sequence types (CST). (XLS)
