# Supplementary figures and images for: CRISPR-cas Subtype I-Fb in Acinetobacter baumannii: Evolution and Utilization for Strain Subtyping
Source: PLoS One. 2015 Feb 23;10(2):e0118205. doi: 10.1371/journal.pone.0118205 (PMC4338279; doi:10.1371/journal.pone.0118205)

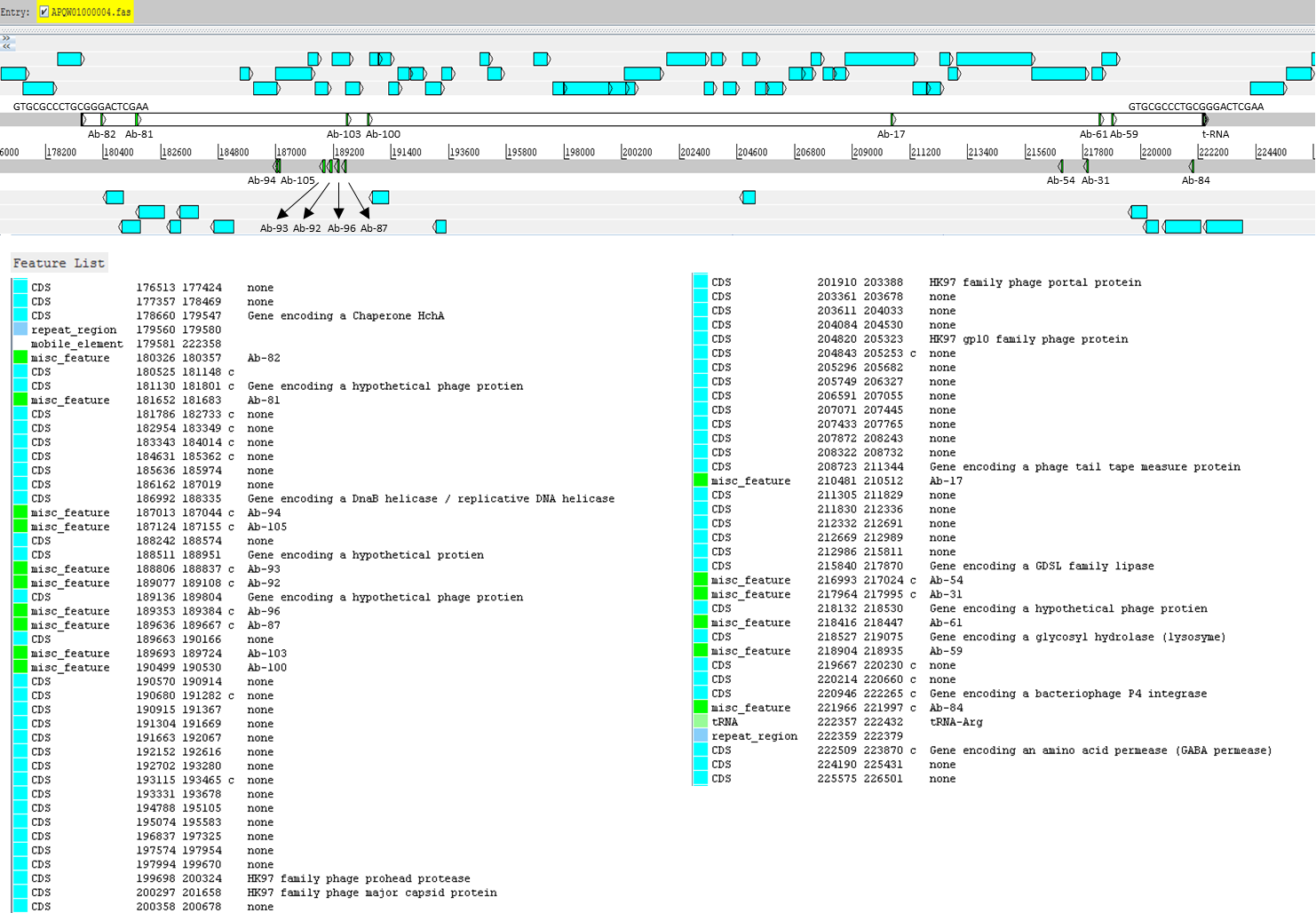

Supplement: S1 Fig — The prophage, 42778-bp long, was located on the genome of A. baumannii NIPH 527 (APQW01000004: 179581–222358). The prophage was shown as a white box on the graph and described as a “mobile_element” in the feature list. Genes and open reading frames were shown as blue arrows, with the arrowheads indicating the direction of transcription. Proto-spacers were presented as labeled green arrows, with the arrowheads indicating the direction of their integration as spacers in the CRISPR arrays. The prophage was surrounded by two identical 20-bp repeat regions, for which the sequences were indicated on the graph. The map was created using Artemis (http://www.sanger.ac.uk/resources/software/artemis/). (TIF) [file pone.0118205.s001.tif]

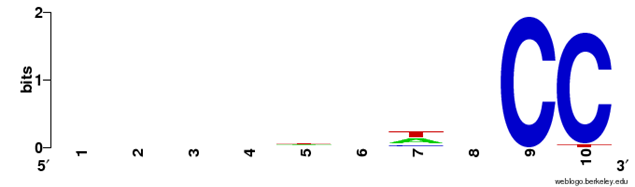

Supplement: S2 Fig — The sequence logo was created based on an alignment of 10-bp sequences adjacent to 63 proto-spacers targeted by the CRISPR-Cas subtype I-Fb system of A. baumannii. The alignment and the logo were created using WebLogo (http://weblogo.berkeley.edu/logo.cgi). The logo consisted of stacks of letters, with a maximum height of 2 bits. The height of letters within each stack reflects the relative frequency of the corresponding nucleotide at that position. The alignment defined CC as the proto-spacer adjacent motif (PAM) for the CRISPR-Cas subtype I-Fb machinery of A. baumannii. (TIF) [file pone.0118205.s002.tif]

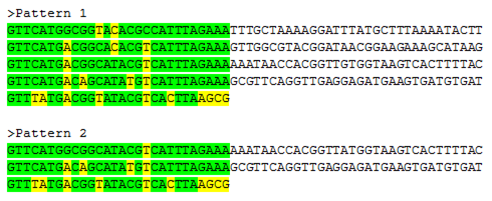

Supplement: S3 Fig — Pattern 1 was detected in all isolates from CC1, ST38, ST428, ST505, and ST519, and isolate 4190 from CC25. Pattern 2 was detected in all isolates from CC25, except for strain 4190, and in the isolates from ST113, ST126, ST12, and ST427. Preserved and degenerated nucleotides of the direct repeats were marked in green and yellow, respectively. (TIF) [file pone.0118205.s003.tif]

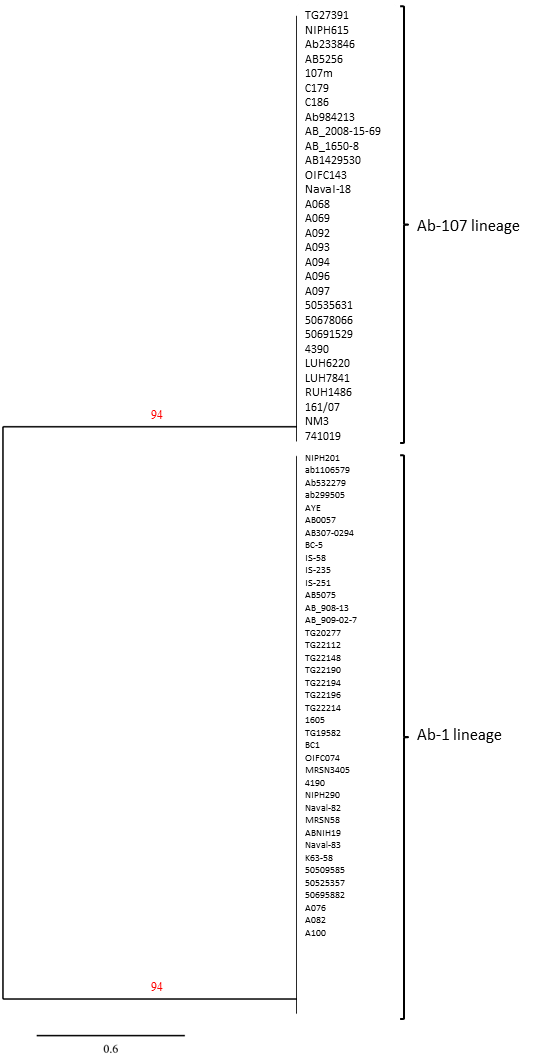

Supplement: S4 Fig — The tree was based on aligned nucleotide sequences of 101 bp from 69 isolates of Acinetobacter baumannii. MUSCLE, Gblocks, PhyML, and TreeDyn were used for nucleotide alignment and tree construction. One hundred bootstraps were used for bootstrap analysis. Branch support values were displayed in %. Isolates of linages Ab-1 and Ab-107 were indicated by braces. (TIF) [file pone.0118205.s004.tif]
